# Supplementary material for: Acceptance and compliance with micronutrient powder (MNP) among children aged 6–23 months in northern Nigeria
Source: PLOS Glob Public Health. 2022 Oct 17;2(10):e0000961. doi: 10.1371/journal.pgph.0000961 (PMC10022258; doi:10.1371/journal.pgph.0000961)
Supplement: S1 Dataset — (ZIP) [file pgph.0000961.s008.zip › S8_Dataset/Direct Observations.zip!/Direct Observations/230615_K_J_SA_1_DO.pdf]

1.1.1 Instrument F –Direct Observation Form

<<Instrument F Direct Observation Form>>
**PI Name:** PragyaMathema,Stephen Kodish<br>**Study Title:** Formative research to develop an infant and young child feeding (IYCF) intervention with micronutrient powder (MNP) for children aged 6 – 23 months<br>**PI Version/Date:** Version1.0/January 24, 2015

Date: 23/06/15Location: Arausaya/Jega

Data Collector: Samira Adamu

Main goals of this direct observation are to answer the following questions:
1. In this household, what would be the best way(s) to give nutritional supplements to young children? Why?<br><br>2. In this household, describe the food sharing that you observe throughout the meal. Are there any predictable patterns?<br><br>3. In this household, who is favored and how is this favoritism expressed during meal preparation and eating?

Household Composition Table (Fill out as you do data collection)

INITIALS	SEX (M/F)	AGE (estimated)	Relationship to household	Description (not necessary)
BB	F	21months	Youngest child	Baby girl with pink earrings and plump
MB	F	18years	Mother	Medium height, dark with yellow and pink wrapper
FB	M	22years	Father	Tall dark wearing sky blue clothes
B1	M	5years	Cousin	Wearing green and milk trousers
BB2	F	2years	Cousin	Slim baby girl with black rope tied to the neck
DL	F	20years	Senior daughter-in law	Yellow and brown wrapper with green
CDL	F	25years	Wife of uncle	Yellow, orange wrapper and pregnant
SB	F	4years	1st child	Wearing ash skirt and blue beaded necklace

PARTICIPANT ID: __________

TIME	ACTOR INITIALS	RECIPIENT INITIALS	WHAT HAPPENED	FOOD	Reactivity
8:00	MB	BB	Breastfeeding the baby	Breast milk	
8:05	MB	BB	Breastfeeding the baby	Breast milk	
8:10	MB	BB	Breastfeeding the baby	Breast milk	
8:15	FB	BB	Playing with the child		
8:20	MB	BB	Wearing pants for the child		Wearing a pant for the baby which is unusual
8:25	BB		Playing with a toy		
8:30	BB		Playing with children		
8:35	BB		Going out with cousin		
8:40	BB		The child is crying		
8:45	MB	BB	Carrying the child to another side of the house		
8:50	BB	BB	Eating tuwo and miyan kadi in uncle's house	Tuwon dawa	
8:55	BB	BB	Eating tuwo and miyan kadi		
9:00	BB	BB	Eating tuwo and miyan kadi		
9:05	BB	BB	Eating tuwo and miyan kadi in uncle's house		
	MB		Processing millet to remove shaft	Millet	
	DL		Processing millet to remove shaft		
9:10	MB		Fetching water from well		
	MB	BB	Giving the child water	Water	

TIME	ACTOR INITIALS	RECIPIENT INITIALS	WHAT HAPPENED	FOOD	Reactivity
9:15	BB	BB	Continued eating tuwo and miyan kadi in uncle's house	Tuwon millet	
9:20	MB		Processing millet to remove shaft	Millet	
	DL		Processing millet to remove shaft	Millet	
9:25	BB	BB	Eating tuwo and miyan kadi		
	MB		Processing millet to remove shaft	Millet	
	DL		Processing millet to remove shaft	Millet	
9:30	BB		Playing with ash and sand		
	MB		Pounding the millet	Millet	
9:35	MB	BB	Backing the baby		
	DL		Still processing the millet to remove shaft	Millet	
9:40	MB	BB	Backing the baby and removing the millet to remove shaft	Millet	
9:45	MB	BB	Backing the baby and removing the millet to remove shaft	Millet	
	DL		Processing more millet to remove shaft	Millet	
9:50	MB	BB	Backing the baby and draining the washed processed millet to dry	Millet	
9:55	MB	BB	Backing the baby and washing plates, bowls and pots	Millet	
	DL		Pounding millet to flour	Millet	
10:00	BB		Sleeping on the bed		
	DL		Pounding millet to flour	Millet	
	MB		Washing plates, bowls and pots		

TIME	ACTOR INITIALS	RECIPIENT INITIALS	WHAT HAPPENED	FOOD	Reactivity
10:05	BB		Sleeping on the bed		
	MB		Washing plates and bowls		
10:10	BB		Sleeping on the bed		
	DL		Pounding millets to flour	Millet	
	MB		Draining her washed plates and bowls		
10:15	BB		Sleeping on the bed		
	MB		Sweeping the compound		
	DL	BB2	Backing the baby and pounding millets to flour	Millet	
10:20	BB		Still sleeping on the bed		
10:25	BB		Sleeping on the bed		
	MB		Pounding millet into flour	Millet	
	DL		Pounding millet into flour	Millet	
10:30	BB		Sleeping on the bed		
	ML		Checking on I and the child		
	MB&DL		Pounding millet to flour	Millet	
10:35	BB		The child is sleeping		
10:40	MB		Pounding to the millet to powder form	Millet	
10:45	BB		Sleeping in the bed		
10:50	BB		Crying in the bed		
10:55	MB	BB	Carrying the baby and breastfeeding the child	Breast milk	
	MB		Sieving the fine grounded millet	Millet	

TIME	ACTOR INITIALS	RECIPIENT INITIALS	WHAT HAPPENED	FOOD	Reactivity
			flour		
	DL		Pounding millet to flour	Millet	
11:00	MB	BB	Breastfeeding the child while sitting on the floor	Breast milk	
11:05	MB	BB	Breastfeeding the child while sitting on the floor	Brest milk	
11:10	MB	BB	Breastfeeding the child while sitting on the floor	Brest milk	
11:15	MB		Sieving the grounded flour to remove th coarse particle		
	DL		Pounding the millet		
	BB		Playing with sand	Millet	
11:20	BB		Playing with sand		
	MB		Sieving the millet flour	Millet	
11:25	BB		Playing with sand on the floor		
11:30	MB	BB	Breastfeeding the child	Breast milk	
11:35	MB	BB	Giving the child water		
11:40	MB	BB	Breastfeeding the child	Breast milk	
11:45	MB	BB	Breastfeeding the child	Breast milk	
11:50	BB		Playing on the bed		
	MB		Sleeping on the bed beside the child		
11:55	MB		Sleeping on the bed beside the child		
	BB		Playing on the bed		
12:00	MB	BB	Breastfeeding the child	Breast milk	
	DL		Pounding millet again	Millet	

TIME	ACTOR INITIALS	RECIPIENT INITIALS	WHAT HAPPENED	FOOD	Reactivity
12:05	MB	BB	Breastfeeding the child both of them are sleeping on the bed	Breast milk	
		DL	Sieving the millet flour	Millet	
12:10	MB	BB	Breastfeeding the child both of them are sleeping on the bed	Breast milk	
12:15	MB	BB	Coming out of the room		
12:20	MB	BB	Breastfeeding the child	Breast milk	
12:25	DL	MB	Gisting with the mother		
12:30	MB	BB	Carrying the child		
	MB		Covering sieved flour in a calabash		
12:35			Playing beside her mother		
12:40	ML		Coming into their side		
	MB		Carrying the child		
	DL		Spreading the millet flour in a calabash	Millet	
12:45	ML		Playing with the child		
	SB	BB	Playing with the child		
12:50	BB		Playing on her mother's laps		
12:55	MB		Selling henna (lalle) to some girls		
	DL	BB2	Backing the child		
1:00	MB	BB	Giving the child water		
1:05	MB	BB	Giving the child biscuits	Biscuits	
1:10	BB	BB	Eating biscuits	biscuits	
	MB	BB	Feeding the child biscuits	biscuits	

TIME	ACTOR INITIALS	RECIPIENT INITIALS	WHAT HAPPENED	FOOD	Reactivity
1:15					
1:20	MB	BB	Breastfeeding the child while sitting on a stone.	Breast milk	
1:25	MB	BB	Breastfeeding the child while sitting on a stone.	Breast milk	
1:30	BB		Playing around the house alone as the mother is performing ablution.		
1:35	MB	BB	Breastfeeding the child on the stool where she performed ablution.	Breast milk	
1:40	MB	BB	Breastfeeding the child on the stool where she performed ablution	Breast milk	
1:45	BB		Playing alone in the compound while the mother is praying.		
1:50	MB	BB	Carrying the child to the senior daughter in-law's side		
1:55	MB		Pounding millet to flour as the child is with the grandmother.	Millet	
	DL		Pounding millet to flour	Millet	
2:00	MB		Sieving the millet flour to remove coarse grains.	Millet	
	BB		Standing beside the mother as she sieve's flour.		
2:05	MB		Pounding the coarse flour after sieving to extract more flour	Millet flour	
	BB		Sitting close to the mother as she sieve's the flour.		
2:10	MB	BB	Still pounding the coarse flour while carrying the baby.	Millet flour	
	BB		Playing around the mother		
2:15	MB		Sieving the millet flour	Millet flour	
	BB		Wearing mother's shoe		
2:20	MB		Pounding the coarse flour after sieving to extract more flour	Millet flour	
	MB		Making a paste of the flour with	millet	

2:25			water to form a mixture of the millet flour and water.		
2:30	MB		Covering the already mixed paste properly		
	BB		Following the mother as she is covering		
2:35	MB	BB	Breastfeeding the child on the floor in front of her room.	Breast milk	
2:41	MB	BB	Breastfeeding the child on the floor in front of her room.	Breast milk	
2:41	BB		Going to grand mother's side alone.		
2:45	SB	BB	Playing with the child		
2:50	SB	BB	Playing with the grandmother and sister		
2:50	SB	BB	Drinking kunu millet, she gave the child and continued drinking	Kunun millet	
2:55	BB	BB	Drinking kunu by herself	Kunun millet	
3:00	BB	BB	Drinking kunun millet by herself	Kunun millet	
3:05	MB	BB	Breastfeeding the child at the grandmother's side in the house	Breast milk	
3:10	MB	BB	Breastfeeding the child while sitting on the mat.	Breast milk	
3:15	MB		Processing sorghum for the tuwo by the removing the shaft.		
	BB		Standing close to the mother as she process the sorghum.		
3:20	MB		Processing the sorghum for tuwo		
	BB		Playing with her sister		
3:25	BB		Sitting with the grandmother in her side.		
	MB		Processing sorghum to remove shaft.		
3:30	MB		Processing another sorghum to remove shaft by gentle pounding of the grains against tomorrow.	Sorghum	

3:35	BB		Playing with the sister		
	MB&DL		Processing the sorghum to remove the shaft from the grain		
3:40	SB	BB	Sister trying to remove charcoal from the sister's mouth		
	ML	BB	Removing the charcoal from the mouth.		
	MB		Processing to remove shaft from the sorghum.		
	DL		Also processing to remove sorghum shaft from the sorghum.		
3:45	MB	BB	Breastfeeding the baby while sitting on the floor.	Breast milk	
	DL	BB2	Giving her meat to eat.		
3:50	BB		Playing with a stick		
	B1	B1	Taking/drinking kunu by herself.	Kunun millet	
3:55	MB	BB	Breastfeeding the baby while standing.	Breast milk	
4:05	MB	BB	Breastfeeding the baby while standing.	Breast milk	
4:10	MB	BB	Breastfeeding the baby while standing.	Breast milk	
4:15	MB		Processing sorghum to remove shaft by gentle pounding.	Sorghum	
	BB		Playing with a stick		
4:20	MB		Processing sorghum to remove shaft by gentle pounding.	Sorghum	
	BB		Playing with stick		
4:25	BB&SB		Drinking kunu millet	Kunun millet	
	MB		Processing sorghum to remove shaft	Sorghum	
4:30	MB		Washing the processed sorghum and draining it to dry before pounding	Sorghum	

	BB		Stooling on the floor		
4:35	BB&SB		Playing together as mother is praying		
4:40	BB,SB&B1		Playing together with sticks		
	MB		Spreading the drained sorghum to air dry well	Sorghum	
	DL		Taking a nap		
	ML		Sweeping animal waste(feaces)		
4:45	MB	BB	Breastfeeding the child	Breast milk	
4:50	MB	BB	Breastfeeding the child	Breast milk	
4:55	BB		She is crying and the mother is in the toilet.		
5:00	MB	BB	Giving her water	Water	
5:05	MB	BB	Breastfeeding the child	Breast milk	
5:10	BB	BB2	Playing with her on the mat		
	MB		removing dirty particles from the dried sorghum		
5:15	DL		Washing plates and bowls		
	BB		The baby is crying		
	MB		Applying charcoal to an old wound		
5:20	BB		Playing with the charcoal		
5:25	MB	BB	Breastfeeding the child while sitting on the mat.	Breast milk	
5:30	MB	BB	Breastfeeding the child le sitting on the mat.	Breast milk	
5:35	MB		Adding a sachet of MNP to cold kunun which was brought from a neighbor’s house	Kunun millet	
5:40	MB	BB	Forcibly feeding the child with MNP mixed portion as she want to take	Kunun millet	

			the kunu herself. She finished the kunun mixed with the MNP although it poured on her body.		
5:45	BB		Crying because she was forced to take the kunu	Kunun millet	
5:50	BB		Drinking kunu	Kunun millet	
5:55	BB		Playing with sticks		
6:00	BB2		Eating bulla and miyan kadi	Bulla and miyan kadi	
	BB		Playing with a stick		

Primary Questions (Complete <u>after</u> you complete data collection):

From what you have seen observing THIS HOUSEHOLD, please answer the following questions in detail:

Question	Write your <u>detailed</u> response here
1. In this household, what would be the best way(s) to give nutritional supplements such as an MNP to young children to ensure compliance?<br><br>Why?	The mother is the best way to give this supplement.<br><br>I observed that the child is always with the mother and she bearly stays away from the mother. Because of this, I suggest the mothe is the best way to ensure compliance
2. In this household, who is favored during meal preparation or feeding?	As far as I am concerned, there is no favoritism expressed among the family members especially the children and throughout my stay all the children did not eat together, except for the first and second child when they took kunu together and the first child gave the baby once.

How is this favoritism expressed?	
3. How could we promote the MNP supplement for this family that would attract their attention?<br><br>Who would they listen to?	It should be promoted by giving the caregivers along with eating utensils and/some incentives (like biscuits).because there is inequality among the daughter in -laws. Also there was more preference on the care giver whose child was 2 years old. She is given more attention and favored her children in terms of food. There is envy and jealousy expressed by the senior daughter in-law over the caregiver to whom MNP was issued.<br><br>They will listen to their husbands because the women are answerable to their husbands. Maybe the mother in-law although she is treating the in-laws like friends and as such I don't know if they will be answerable to her.
4. In this household, what would be the best ways to promote the MNP but limit how much it would be shared?<br><br>Why?	There should be proper sensitization on the usage, direction of usage as well as importance of the MNP and the need not to share. The little children eat separately so there will be no sharing involved during eating.<br><br>There are four caregivers, so if each caregiver is given the MNP because three of them have children of 6-23months, then probably, sharing would be limited and it should be presented to them more or less like medicine. With this, it will let them not to allow other children use it.
5. What other issues and concerns did you see with this family that are important	Improper hygiene is practiced in the house as the farm animals could eat directly from the plates of the children. Hence, if MNP is administered into the child's food, and any of the farm animals

for us to consider with regard to introduction of an MNP?<br><br>Why?	accidentally feed on the food, since the little children are allowed to feed themselves, then the aim is not achieved and sharing is not hindered.<br><br>Also the consistency and temperature of the food served with MNP needs to be explained properly. Because, she added MNP to cold kunu which was insoluble and I think this made the child not to drink the kunu.
6. Based on your experience observing this child today, do you believe reactivity negatively impacted your ability to see the food-related behaviors of the child?<br><br>Please explain your answer in detail.	No I don't think so, because the child was allowed to eat just as she does even in my absence.<br><br>Because, when she eating tuwo and miyan kadi in uncle's house, I just entered without them knowing I was coming so she exhibited her actual eating behavior with no reactivity. They were very free with me so there was no reactive behavior recorded in the household concerning eating behavior.
